# Clinical manifestations of tension pneumothorax: protocol for a systematic review and meta-analysis

**DOI:** 10.1186/2046-4053-3-3

**Published:** 2014-01-04

**Authors:** Derek J Roberts, Simon Leigh-Smith, Peter D Faris, Chad G Ball, Helen Lee Robertson, Christopher Blackmore, Elijah Dixon, Andrew W Kirkpatrick, John B Kortbeek, Henry Thomas Stelfox

**Affiliations:** 1 Department of Surgery, University of Calgary and the Foothills Medical Centre, 1403-29th Street NW, T2N 2T9, Calgary, Alberta, Canada; 2 Department of Community Health Sciences (Divisions of Epidemiology), University of Calgary, 3280 Hospital Drive NW, T2N 4Z6, Calgary, Alberta, Canada; 3 Defence Medical Services, Royal Navy and Royal Infirmary Edinburgh, 51 Little France Cres, Old Dalkeith Road, EH16 4SA, Edinburgh, UK; 4 Alberta Health Services - Research Excellence Support Team, 1403-29th Street NW, T2N 2 T9, Calgary, Alberta, Canada; 5 Department of Oncology, University of Calgary and the Foothills Medical Centre, 1403-29th Street NW, T2N 2 T9, Calgary, Alberta, Canada; 6 Regional Trauma Services, the Foothills Medical Centre, 1403-29th Street NW, T2N 2 T9, Calgary, Alberta, Canada; 7 Health Sciences Library, Health Sciences Centre, University of Calgary, 3330 Hospital Drive NW, T2N 4 N1, Calgary, Alberta, Canada; 8 Department of Critical Care Medicine, University of Calgary and the Foothills Medical Centre, 3134 Hospital Drive NW, T2N 5A1, Calgary, Alberta, Canada; 9 Department of Medicine, University of Calgary and the Foothills Medical Centre, 3134 Hospital Drive NW, T2N 5A1, Calgary, Alberta, Canada

**Keywords:** Tension pneumothorax, Clinical manifestations, Systematic review, Meta-analysis, Observational study, Rare conditions, Case report, Case series, Case study

## Abstract

**Background:**

Although health care providers utilize classically described signs and symptoms to diagnose tension pneumothorax, available literature sources differ in their descriptions of its clinical manifestations. Moreover, while the clinical manifestations of tension pneumothorax have been suggested to differ among subjects of varying respiratory status, it remains unknown if these differences are supported by clinical evidence. Thus, the primary objective of this study is to systematically describe and contrast the clinical manifestations of tension pneumothorax among patients receiving positive pressure ventilation versus those who are breathing unassisted.

**Methods/Design:**

We will search electronic bibliographic databases (MEDLINE, PubMed, EMBASE, and the Cochrane Database of Systematic Reviews) and clinical trial registries from their first available date as well as personal files, identified review articles, and included article bibliographies. Two investigators will independently screen identified article titles and abstracts and select observational (cohort, case–control, and cross-sectional) studies and case reports and series that report original data on clinical manifestations of tension pneumothorax. These investigators will also independently assess risk of bias and extract data. Identified data on the clinical manifestations of tension pneumothorax will be stratified according to whether adult or pediatric study patients were receiving positive pressure ventilation or were breathing unassisted, as well as whether the two investigators independently agreed that the clinical condition of the study patient(s) aligned with a previously published tension pneumothorax working definition. These data will then be summarized using a formal narrative synthesis alongside a meta-analysis of observational studies and then case reports and series where possible. Pooled or combined estimates of the occurrence rate of clinical manifestations will be calculated using random effects models (for observational studies) and generalized estimating equations adjusted for reported potential confounding factors (for case reports and series).

**Discussion:**

This study will compile the world literature on tension pneumothorax and provide the first systematic description of the clinical manifestations of the disorder according to presenting patient respiratory status. It will also demonstrate a series of methods that may be used to address difficulties likely to be encountered during the conduct of a meta-analysis of data contained in published case reports and series. PROSPERO registration number: CRD42013005826.

## Background

Tension pneumothorax, often defined as hemodynamic compromise in a patient with an expanding intrapleural air mass [1], is an uncommon yet potentially catastrophic clinical diagnosis most frequently encountered in pre-hospital, Emergency Department, and Intensive Care Unit (ICU) settings [2-7]. Although a valid estimate of the incidence of tension pneumothorax remains to be determined, this condition has been suggested to occur among 5% of major trauma patients managed in the pre-hospital environment and 1% to 3% of adult ICU patients [2,4,7,8]. In one retrospective cohort study, the adjusted risk of death among mechanically ventilated patients was reported to be approximately 38 times higher among those who developed a tension pneumothorax as compared to those who did not [9].

As tension pneumothorax is associated with substantial mortality, the Advanced Trauma Life Support (ATLS®) guidelines recommend that attempts be made to diagnose this condition during the initial minutes of trauma patient assessment [10]. Moreover, possibly because waiting for a chest radiograph has been associated with an increased risk of death among mechanically ventilated patients [11], most authorities recommend emergent treatment with needle or tube thoracostomy before radiographic confirmation when the condition is first suspected [1,7,10,12-16]. Thus, prehospital providers and physicians utilize classically described clinical manifestations to diagnose tension pneumothorax. These have most frequently been reported to include hemodynamic compromise (hypotension or cardiac arrest) in conjunction with signs suggestive of a pneumothorax (hypoxia, respiratory distress, absent unilateral breath sounds on auscultation) and mediastinal shift (tracheal deviation and jugular venous distention) [7,17].

### Potential for divergent clinical manifestations among patients with a tension pneumothorax according to presenting respiratory status

Unfortunately, although tension pneumothorax is commonly considered to be a clinical diagnosis, available textbooks, narrative review articles, and guidelines differ in their descriptions of its clinical manifestations [1,7,12-15]. This finding is concerning as delayed or even missed diagnoses have been reported among patients lacking classically described findings of the disorder [7,18,19]. Moreover, possibly because the clinical manifestations of tension pneumothorax have never been systematically reviewed, many sources appear to have derived their descriptions of the signs and symptoms of the disorder from the pathophysiologic processes observed in original canine models of the disorder [17,20,21]. These models, which continue to inform medical literature and teaching even today, suggest that the clinical manifestations of tension pneumothorax result from: 1) loss of ipsilateral negative intrapleural pressure, 2) contralateral mediastinal shift and compression of the intrathoracic vena cavae and/or angulation of the caval-atrial junction, and 3) reduced venous return to the heart leading to cardiovascular collapse [17].

Although commonly described among the medical literature, the generalizability of the above pathophysiologic mechanisms to humans is challenged by anatomical differences between dogs and humans [17,21]. Instead of being rigid and relatively fixed, the mediastinum of dogs is compliant and does not tolerate development of a pressure gradient between contralateral pleural spaces [17,21]. Thus, subsequent models of tension pneumothorax that utilized animals with a mediastinum similar to that of an adult human (goats, pigs, and sheep) demonstrated markedly different pathological mechanisms of the disease, which appeared to vary according to the respiratory status (and possibly the level of alertness or consciousness) of the animal [7,17,22,23]. Most importantly, these studies demonstrated that because awake or lightly sedated and spontaneously breathing animals utilize a number of compensatory mechanisms during evolution of a tension pneumothorax, they may be relatively protected from development of hypotension until the pre-terminal stages of the disorder [17,22].

These compensatory mechanisms, which may also occur among awake and spontaneously breathing humans, include a progressive tachycardia, an increasing respiratory rate and tidal volume, and increasingly negative contralateral chest excursions [7,17,22]. Methods by which these mechanisms may maintain arterial blood pressure during tension pneumothorax include: 1) incomplete transmission of ipsilateral pneumothorax-related pressure to the mediastinum and contralateral hemithorax; 2) maintenance of cardiac venous return through rising spontaneous respiratory effort resulting in increasingly negative contralateral intrathoracic pressures during inspiration; and 3) a significant increase in heart rate due to baroreceptor reflexes and/or the effects of catecholamines released onto the heart [22]. Thus, studies of lightly sedated, spontaneously breathing goats and sheep observed the pathophysiology of tension pneumothorax to involve progressive atelectasis resulting in pulmonary arterial shunting, worsening respiratory failure, and death from progressive hypoxemia rather than cardiovascular causes [17,22]. However, in studies of anesthetized pigs receiving positive pressure ventilation (which are likely incapable of mounting a substantial compensatory response due to the effects of sedation and/or substantially elevated inspiratory pressures), a significant and rapid decline in arterial pressure occurred at induced pneumothorax volumes of approximately 57% total lung capacity (followed by cardiovascular collapse at 94% total lung capacity) [24].

Interestingly, some authors have recently highlighted that a number of observational studies and case reports and series now exist that appear to suggest that adults with a tension pneumothorax who are breathing unassisted (that is, breathing spontaneously and not receiving positive pressure ventilation) often develop respiratory symptoms and signs without hemodynamic compromise [7]. These authors and others have also outlined several additional differences that may have clinical importance for the recognition and subsequent treatment of the disorder. First, rather than the initial development of cardiac arrest among those with a tension pneumothorax, these authors suggest that patients who are breathing unassisted may more frequently first develop respiratory arrest, possibly due to respiratory center depression as a result of hypoxemia [25]. Second, while hemodynamic decompensation or cardiac arrest may develop within minutes of pneumothorax onset among patients who are receiving positive pressure ventilation (as has been classically described for this condition), these authors have also suggested that respiratory arrest may not occur for hours to days among those who are breathing unassisted [7,26,27]. If supported by available evidence, these differences could suggest that diagnosis of tension pneumothorax in patients who are breathing unassisted may more appropriately be based on respiratory clinical manifestations, and that earlier diagnosis and more appropriate treatment among these patients may result in improved patient outcomes. An overview of the hypothesized differences in clinical manifestations of tension pneumothorax according to the presenting respiratory status of the patient that have been suggested based largely on animal study data are presented in Table 1.

**Table 1:** **Suggested differences in clinical manifestations among patients with a tension pneumothorax stratified by presenting respiratory status **[1,7,17,22,25]

**Respiratory status**	**Predominant signs and symptoms**	**Arterial blood pressure**	**Method of arrest**	**Time from presentation or pleural injury to arrest**	**Rationale**
Breathing unassisted	Chest pain, dyspnea, respiratory distress, tachypnea, hypoxia and/or increased oxygen requirements, increased respiratory effort and contralateral respiratory excursions, tachycardia	Normal until respiratory arrest or development of decreased level of consciousness (that is, until compensatory mechanisms fail)	Respiratory	Hours	Compensatory mechanisms to progressively increasing ipsilateral pneumothorax size maintain arterial blood pressure until the pre-terminal stages of the disorder
Positive pressure ventilation	Hypoxia and/or increased oxygen requirements, tachycardia, hypotension, and cardiac arrest	Substantially decreased from normal	Cardiac	Minutes	Absence of compensatory mechanisms to progressively increasing ipsilateral pneumothorax size allow for a rapid and significant decline in arterial blood pressure

### Study rationale and objectives

While the above suggested differences in clinical manifestations among patients with a tension pneumothorax may have clinical importance, they remain largely based on narrative or non-systematic syntheses of the available clinical data and therefore could be accounted for by selection bias [1,7]. To our knowledge, only six non-systematic or narrative reviews on tension pneumothorax have been published between the years 1956 and 2013 [1,7,13,14,28,29], and only two of these [1,7] suggested that the clinical manifestations of tension pneumothorax may differ according to the respiratory status of the patient. The absence of a systematic review on this topic may relate to the perceived lack of relevant observational data given that tension pneumothorax is difficult to study (given that it presents acutely, is relatively uncommon and life-threatening, and requires immediate treatment). Observational studies of patients receiving positive-pressure ventilation are likely particularly difficult to conduct as mechanically ventilated patients have been reported to manifest hemodynamic instability within minutes of an observed change in clinical status [30-35].

Likely as a result of these difficulties, although several retrospective observational studies exist that report data on the clinical manifestations of tension pneumothorax [4,11,36-41], the majority of original information on this topic is contained in published case reports and small case series. In our initial scout or feasibility searches that were conducted during preparation of this systematic review protocol, we identified over 200 case reports or small case series describing the clinical presentation of patients with a tension pneumothorax. Importantly, these case reports/series appear to frequently report the respiratory status of the described study patient(s) and provide detailed information regarding the associated signs and symptoms of tension pneumothorax, including findings observed on invasive intravascular catheters, mechanical ventilators, and echo- and electrocardiograms.

As tension pneumothorax is frequently a difficult clinical diagnosis encountered in emergent situations [42], the primary objective of this systematic review and meta-analysis is to utilize the existing world literature (including that from published case reports and series) on tension pneumothorax to describe and contrast the clinical manifestations of the disorder among patients receiving positive pressure ventilation versus those who are breathing unassisted. A secondary objective is to determine if a difference exists in the time to arrest or requirement for thoracic decompression between these two groups. The study protocol described herein will demonstrate methods for a systematic review and meta-analysis of clinical manifestations data that could potentially be used in future studies to better characterize the signs and symptoms observed among patients with an uncommon, rare, or acutely life-threatening condition. Ultimately, results of this work will provide the first systematic description of all currently available, published data on the clinical manifestations of tension pneumothorax. These data will be used to better inform health care providers and therefore may contribute to an improved understanding of the appropriate clinical diagnosis and treatment of this life-threatening condition.

## Methods/Design

### Protocol preparation and registration

Methods for the inclusion and analysis of articles have been developed according to recommendations from the Meta-analysis of Observational Studies in Epidemiology proposal [43], the Preferred Reporting Items for Systematic Reviews and Meta-analyses statement [44], and the Cochrane Collaboration [45]. This protocol has been registered in the PROSPERO International Prospective Register of Systematic Reviews (registration number: CRD42013005826; available at http://www.crd.york.ac.uk/PROSPERO/display_record.asp?ID=CRD42013005826#.UkWeRrykCZA).

### Structured clinical question

Is the reported type and/or frequency of clinical manifestations of tension pneumothorax different among adults/adolescents (≥12 years old) or children (<12 years old) who were receiving positive pressure ventilation as compared to those who were breathing unassisted (that is, spontaneously breathing and not receiving positive pressure ventilation)?

### Search strategy

Three investigators (DR, SL-S, JK) developed a preliminary search strategy that was subsequently refined by an information scientist/medical librarian with extensive systematic review experience (HR). Using Ovid, we will search MEDLINE and MEDLINE In-Process & Other Non-indexed Citations, EMBASE, and the Cochrane Database of Systematic Reviews from their first available date without language or publication date restrictions. We will also query PubMed. To identify unpublished or ongoing/recently concluded studies, we will write colleagues and content experts, search personal files, and investigate two clinical trial registries (ClinicalTrials.gov and Current Controlled Trials). In addition, we will use the PubMed ‘related articles’ and Google Scholar ‘cited by’ features and manually search reference lists of included articles and tension pneumothorax review papers identified during the conduct of the search. Authors of articles will be contacted for additional information as necessary. Searches will be updated to within three months of submission of the results of the systematic review for peer review.

In MEDLINE, we will search the exploded Medical Subject Heading (MeSH) term ‘Pneumothorax’. We will also search the text words ‘tension’, ‘tension physiology’ , ‘expanding’ , ‘needle thoracentesis’ , ‘thoracentesis’ , ‘needle thoracostomy’ , ‘needle aspiration’ , ‘chest decompression’ , ‘thoracic decompression’ , and ‘needle decompression’ , and then combine these through use of the Boolean operator ‘OR’. This key word search (or tension search theme) will subsequently be combined with our pneumothorax MeSH term query using the Boolean operator ‘AND’ in order to create a unique tension pneumothorax search theme. This search theme will then be combined with the key term ‘tension pneumothora*’ using the Boolean operator ‘OR’. A similar electronic search strategy will be used to investigate all remaining databases (see Table 2 for a detailed description of our database search strategies).

**Table 2:** Details of electronic bibliographic database search strategies

**Ovid MEDLINE and MEDLINE In-Process & Other Non-indexed Citations and the Cochrane Database of Systematic Reviews**	**Ovid EMBASE**
1. Exp Pneumothorax/	1. Exp Pneumothorax/
2. Tension pneumothora$.mp. [mp = title, original title, abstract, name of substance word, subject heading word, unique identifier]	2. Tension pneumothora$.mp. [mp = title, original title, abstract, name of substance word, subject heading word, unique identifier]
3. Tension.mp	3. Exp Tension/
4. Tension physiolog$.mp	4. Tension.mp
5. Expanding.mp	5. Tension physiolog$.mp
6. Needle thoracentesis.mp	6. Expanding.mp
7. Thoracentesis.mp	7. Needle thoracentesis.mp
8. Needle thoracostomy.mp	8. Thoracentesis.mp
9. Needle aspiration.mp	9. Needle thoracostomy.mp
10. Chest decompression.mp	10. Needle aspiration.mp
11. Thoracic decompression.mp	11. Chest decompression.mp
12. Pleural decompression.mp	12. Thoracic decompression.mp
13. 3 or 4 or 5 or 6 or 7 or 8 or 9 or 10 or 11 or 12	13. Pleural decompression.mp
14. 1 and 13	14. 3 or 4 or 5 or 6 or 7 or 8 or 9 or 10 or 11 or 12 or 13
15. 2 or 14	15. 1 and 14
	1. 2 or 15

‘exp’ denotes that the search will be exploded; ‘$’ indicates that a wild card search will be performed: thus, with the use of this symbol, when ‘pneumothora$’ is searched, both ‘pneumothorax’ and ‘pneumothoraces’ will be searched.

### Review procedure

Independently and in duplicate, two investigators (DR, CB) will screen citation titles and abstracts and review potentially relevant articles in full. We will consider published observational (cohort, case–control, and cross-sectional) studies and case reports and series that report original data on clinical manifestations of tension pneumothorax for inclusion in the systematic review. Clinical manifestations will be defined as patient-level findings/data, which may be gathered by clinicians during a medical interview or through physical examination, invasive monitoring or treatment equipment (for example, intravascular catheters or mechanical ventilators), and diagnostic studies (for example, echo- and electrocardiograms) [46]. All published observational studies and case reports and series in which tension pneumothorax was diagnosed by the study authors/involved clinicians (and for which data on clinical manifestations were reported) will be eligible for inclusion. Reports of fatal cases will be included if the condition causing death was attributed by the study authors to be a tension pneumothorax and associated with expulsion of air following thoracic decompression or determined by a pathologist to be present on autopsy.

As tension pneumothorax is a syndrome diagnosis without an independent reference-standard diagnostic test [10,16,40], any systematic review of its clinical manifestations may be limited by incorporation bias (whereby the estimation of the frequency of clinical manifestations that may have been incorporated into the diagnosis may bias upward the results [46]). To reduce the risk of this bias, two investigators (DR, CB) will independently determine whether the clinical condition of the study patient(s) presented in each case report aligned with a previously published tension pneumothorax working definition [7,47]. According to this definition, a tension pneumothorax is defined not only by the type of its presenting clinical manifestations, but also according to its response to treatment as one ‘that results in significant respiratory or hemodynamic compromise that reverses [or at least significantly improves] on thoracic decompression alone’ [7,47].

We will exclude observational studies and case reports/series that do not describe ventilation status, as well as those involving patients with diving-related pulmonary barotrauma; a previous contralateral pneumonectomy, traumatic diaphragmatic hernia, or tension pneumopericardium or pneumoperitoneum; as well as those with chronic (as defined by the authors) or loculated pneumothoraces. We will also exclude observational studies and case reports/series of patients undergoing thoracic surgery or laparoscopy at the time of onset of their tension pneumothorax clinical manifestations. All of the above excluded conditions were selected as they represent special, uncommon, or less relevant associated or principal patient conditions, which have the potential to misrepresent the more common clinical manifestations of tension pneumothorax.

Disagreements between investigators regarding the above decisions will be resolved by consensus and, if needed, arbitration by a third investigator. Inter-investigator agreement will be quantified by calculating a kappa (κ) statistic and associated 95% confidence interval (CI) [48].

### Data extraction

Independently and in duplicate, two investigators (DR, CB) will extract data using a pre-designed electronic spreadsheet. These data collection instruments will be piloted on a random selection of five observational studies and 30 case reports/series until reliable data collection can be demonstrated (κ statistic ≥0.75) [48]. All non-English citations will be read in full by an interpreter, and, for those studies and reports/series that satisfy selection criteria for inclusion in the systematic review, data will be extracted only once by this individual. Data extracted from individual articles will include:

1. Study characteristics, including year of publication, country of conduct, and design. The design of the included studies (for example, case–control versus cohort) will be classified using the scheme developed by Oleckno [49]. As it may sometimes be difficult to distinguish between cohort studies and case series, a cohort study will be defined according to the definition proposed by Dekkers as a study in which patients are sampled based on exposure and the occurrence of outcomes was assessed (as an aggregate measure) during a follow-up period [50].

2. Characteristics of the study participants, including the number enrolled as well as their age and gender, pneumothorax etiology (as suggested by the authors or through consensus between investigators), and ventilation status (that is, whether they were receiving positive pressure ventilation or were breathing unassisted), as well as whether a definition of tension pneumothorax was provided and how this condition was specifically defined. We will define positive pressure ventilation as either invasive (for example, via an endotracheal tube and mechanical ventilator) or non-invasive (for example, bag-valve-mask ventilation). Mechanical ventilator settings will also be collected where available.

3. Whether the following clinical manifestations were present before and after thoracic decompression: chest pain, dyspnea or shortness of breath, respiratory distress, subcutaneous emphysema, hypoxia (either arterial oxygen saturation (SpO_2_) <92% or partial pressure of arterial oxygen (PaO_2_) <8 kPa/60 mmHg on room air or requiring supplemental oxygen], tachypnea (respiratory rate ≥20), tachycardia (heart rate ≥100), hypotension (systolic blood pressure ≤ 90 and/or mean arterial pressure ≤ 60 mmHg), jugular venous distention, increased peak inspiratory or airway pressure and/or whether resistance to assisted ventilation was noted by the clinicians managing the patient, any reported relevant invasive hemodynamic (for example, cardiac output or central venous or pulmonary arterial pressures) or respiratory (mechanical ventilatory) measurements, and whether the patient developed a respiratory and/or cardiac arrest (and which occurred first) [1,7]. We will also record the first reported type of cardiac arrest rhythm and the approximate time to cardiac or respiratory arrest (or requirement for thoracic decompression) where available. Finally, we will extract values for respiratory and heart rates; systolic, diastolic, and mean arterial blood pressures; and SpO_2_ or PaO_2_ (as well as the accompanying fraction of inspired oxygen (FIO_2_) that the patient was receiving) at baseline (that is, before any change in clinical status) and before and after thoracic decompression where possible. Through consensus between the two extracting investigators, the clinical manifestations of tension pneumothorax will be extracted as proximal to the author’s description of its diagnosis and/or treatment as possible (that is, immediately prior to diagnosis of the condition by a physician or other healthcare provider). We will accept authors’ definitions for subjective clinical manifestations such as respiratory distress and for the presence or absence of dichotomous clinical manifestations (for example, tachycardia or hypotension) where absolute numbers were not afforded.

4. Whether the following ipsilateral and contralateral chest signs were present before and after thoracic decompression: percussion hyper-resonance, decreased air entry, thoracic hyper- or hypo-expansion, chest wall hyper- or hypo-mobility, and contralateral tracheal deviation [1,7].

5. Whether the following X-ray or computed tomography signs of tension pneumothorax were present before and after thoracic decompression: a large (>50% total lung volume) pneumothorax, tracheal and/or mediastinal shift, increased rib spacing, and/or a flattened ipsilateral hemidiaphragm [29].

6. The initial and subsequent method of thoracic decompression for treatment of tension pneumothorax (needle or tube thoracostomy or another method) and whether these were effective.

7. Any confounding treatments or pathologies that could alter the clinical manifestations of patients with a tension pneumothorax, including administration of antihypertensive or vasopressor medications (or chronic use of antihypertensives); history of hypertension, heart failure, or chronic pulmonary disease; presence of rib fractures, flail chest, pulmonary contusions, or hemothorax or other pleural effusion; pre-existing shock; and decreased level of consciousness (as defined by the authors or reported using a commonly used clinical cutoff on a validated scale (for example, Glasgow Coma Scale (GCS) score ≤13) [51]).

### Risk of bias assessment

The same two investigators (DR, CB) will determine risk of bias among the included studies. Using the recommendations proposed by Richardson and colleagues, we will evaluate: 1) whether the diagnosis of tension pneumothorax was determined using credible criteria that were at least partially independent of the clinical manifestations under study (by examining if the clinical diagnosis was supported by radiographic findings/response to thoracic decompression and whether overlap existed between the utilized diagnostic criteria (where reported) and the reported frequency of clinical manifestations); 2) whether patients were representative of the population of patients with tension pneumothorax (by evaluating the setting from which study patients were recruited, the methods used to identify/exclude patients, and whether any important subgroups may have been excluded); 3) whether clinical manifestations were sought thoroughly and consistently by the study authors (by determining the methods by which clinical manifestations were gathered, and whether this was done similarly for all patients); and 4) whether the estimates of the frequency of reported clinical manifestations were precise (by evaluating the width of the reported or calculated 95% confidence interval around these estimates) [46]. We will also describe the temporality (prospective versus retrospective) of the included cohort studies as well as whether their patient enrollment method was consecutive versus non-consecutive.

Case reports and series will be evaluated as to whether authors provided absolute numbers rather than narrative or subjective descriptions when reporting the presence or absence of hypotension among patients with a tension pneumothorax [52,53]. Moreover, the adequacy of reporting on confounding/modifying and clinical manifestations data among included case reports/series will be evaluated by recording whether information on these variables was either reported or unclear/not reported (that is, not specifically mentioned or described as being absent) in the manuscript [52,53]. Finally, we will quantify the extent of unclear/unreported information (as determined by two independent investigators) among the potentially confounding/modifying and principal outcome variables.

Disagreements in methodological assessments will be resolved by consensus or arbitration by a third investigator (HS).

### Data synthesis

#### Overview of the data synthesis strategy

**Figure 1:**
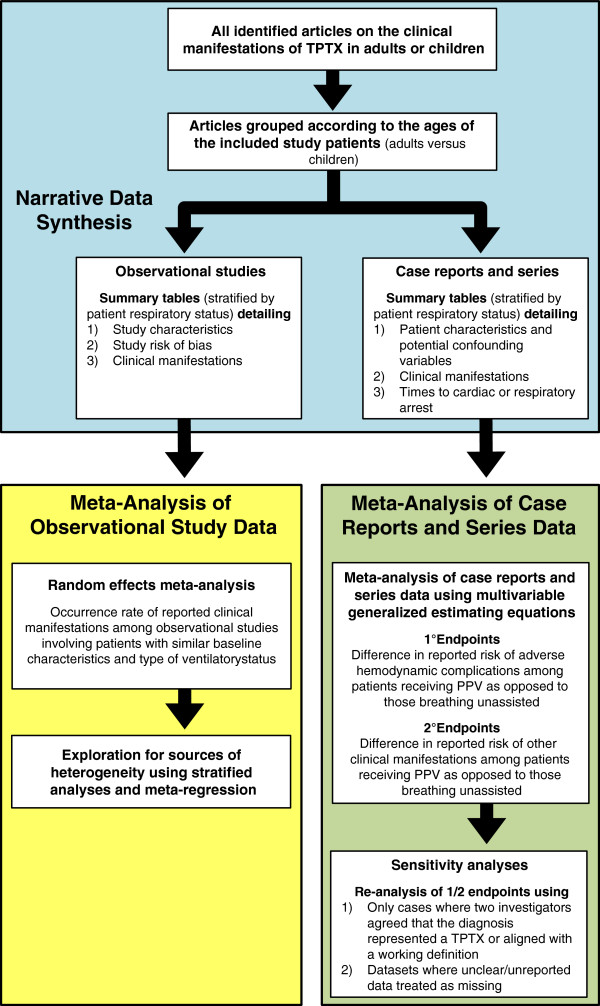
**Overview of the planned strategy for synthesis of data on the clinical manifestations of tension pneumothorax among observational studies and case reports and series.** PPV, positive pressure ventilation; TPTX, tension pneumothorax.

An overview of the planned data synthesis strategy is presented in Figure 1. We will first conduct a narrative synthesis of the systematic review results [54,55]. Where appropriate, this will be followed by a meta-analysis of observational studies and then a separate meta-analysis of case reports and series. Observational studies and case reports and series of patients aged <12 years will be analyzed separately from adolescents/adults (those aged ≥ 12 years) as vital signs vary significantly below 12 years of age, and the clinical manifestations of children likely differ from adults as their mediastinum and thoracic wall are more flexible [10,17].

All clinical manifestations reported among observational studies of patients with a tension pneumothorax who were receiving positive pressure ventilation will be compared to those reported for patients breathing unassisted either indirectly (if only non-comparative studies of patients with one versus another type of ventilation status were available) or directly (if patients with both types of ventilation status existed within studies). This will be done qualitatively and, where possible, quantitatively through use of meta-analysis. Subsequently, summary statistics will be calculated describing the frequency of clinical manifestations reported among case reports and series of patients with a tension pneumothorax stratified by patient ventilatory status. These results will then be compared to those obtained from the observational studies in order to examine for similarities and/or differences. Finally, a meta-analysis of the data reported by case reports and series will be conducted in order to examine whether differences exist in clinical manifestations, including decreases in arterial blood pressures or lower presenting arterial blood pressure values, hypotension, and cardiac arrest, among patients receiving positive pressure ventilation versus those who were breathing unassisted.

#### Narrative synthesis of results

Using recommendations proposed for the conduct of narrative synthesis in systematic reviews [54,55], articles will first be grouped according to the ages of the included study patients (adults/adolescents versus children) and their design (observational studies versus case reports/series). After studies have been appropriately grouped, the principal characteristics of the observational studies (including their design (that is, cohort, case–control, or cross-sectional), year of publication, setting, and a description of the clinical manifestations of the included patients stratified by ventilation status) will be presented in one or more summary tables before any statistical combination of their results is contemplated. Similarly, for case reports and series, the details of the reported patients, including their baseline characteristics (for example, age, gender, and the etiology of their pneumothorax); whether two investigators independently agreed that the diagnosis satisfied the tension pneumothorax working definition; potential confounding conditions or treatments; and clinical, radiological, and invasive hemodynamic or respiratory clinical manifestations will first be presented in summary tables stratified by ventilatory status before any meta-analyses are conducted. We will also present the reported times to cardiac or respiratory arrest (or, where these are unavailable, the time to requirement for thoracic decompression) among patients according to their ventilatory status.

### Statistical analyses

Stata MP version 13.1 (Stata Corp. LP, College Station, TX, U.S.A.) and R version 3.0.1 (available at http://www.r-project.org/) will be used for the conduct of all statistical analyses. Except where mentioned below, two-sided *P* values <0.05 will be considered statistically significant during hypothesis testing.

#### Meta-analysis of observational study data

We will begin our observational study data meta-analysis by calculating individual study estimates of the occurrence rate [56] of clinical manifestations among patients receiving positive pressure ventilation versus those that are breathing unassisted. The occurrence rate will be defined according to Feinstein [56] using the following formula:

$$\mathrm{Occurrence}\;\mathrm{rate}=\frac{\mathrm{Number}\;\mathrm{of}\;\mathrm{reported}\;\mathrm{cases}\;\mathrm{with}\;a\;\mathrm{specific}\;\mathrm{clinical}\;\mathrm{manifestation}}{\mathrm{Total}\;\mathrm{number}\;\mathrm{of}\;\mathrm{reported}\;\mathrm{cases}}$$

The standard error and 95% CI of these estimates will then be determined using the Clopper-Pearson exact binomial method [57].

Among studies with a similar design involving patient populations with similar baseline characteristics, individual study estimates of the occurrence rate of clinical manifestations will be pooled separately stratified by patient ventilatory status. If studies provide the percentage of patients who were receiving positive pressure ventilation, but do not stratify their results by ventilatory status, then those in which <50% of patients were breathing unassisted will be included in the positive pressure ventilation category. Where possible, we will also determine a pooled estimate of the weighted or standardized mean difference in continuously measured clinical manifestations such as systolic or mean arterial pressures between these two patient populations (or the difference in these values between baseline and presentation, where available) [58]. As variability in our pooled estimates beyond chance is expected across studies, these estimates will be calculated using random effects models according to the method proposed by DerSimonian and Laird [58,59].

The pooled estimates obtained from the above calculations will then be compared qualitatively in order to determine if differences exist in the type and/or occurrence rate of clinical manifestations according to the ventilatory status of the study patients. Although we believe that it would be unlikely that any of the available observational studies will report adjusted odds or risk ratios relating the frequency of occurrence of clinical manifestations between patients receiving positive pressure ventilation versus those breathing unassisted, where available these will also be pooled using random effects models [59].

We will examine for evidence of between-study statistical heterogeneity by calculating I^2^ inconsistency and Cochran’s Q statistics (as part of a hypothesis test of heterogeneity) [60,61]. As suggested by Higgins and coworkers [61], we will consider I^2^ statistics ≥25%, 50%, and 75% to represent low, moderate, and high degrees of inter-study heterogeneity, respectively. In the presence of greater than a low degree of between-study heterogeneity, we will conduct subgroup analyses and univariate meta-regression (*P* value <0.10 considered significant given the low power of these tests) in order to explore the influence of sources of clinical and methodological study variation on the meta-analysis results. *A priori* study covariates of interest will include: 1) observational study design (that is, cohort versus case–control or cross-sectional), 2) use of antihypertensive medications or the presence of pulmonary disease among ≥50% of patients who were breathing unassisted, 3) percentage of patients receiving positive pressure ventilation (≥50% versus <50%), and 4) whether the presence of disease was determined using credible criteria that were at least partially independent of the clinical manifestations under study [7,46,47].

#### Meta-analysis of case reports and series data

After stratifying the reported results of case reports and series by the presenting age of the included patient(s) into adults/adolescents versus children, we will begin by examining the distribution of all continuous variables using histograms and measures of central tendency. Normally distributed data will be summarized using means (with standard deviations) and compared using t-tests (with an unequal variance option where appropriate) while skewed data will be summarized using medians (with interquartile ranges) and compared using Mann–Whitney U-tests. Dichotomous data will be summarized using proportions and compared using Fisher’s exact tests.

As stepwise regression procedures [62] may result in biased estimated coefficients and optimistic fits (especially with small sample sizes), our analyses will test the *a priori* hypothesis that patients who are receiving positive pressure ventilation have a higher reported risk of adverse hemodynamic complications. We will also examine whether these reported complications may be confounded or modified by patient age, administration of antihypertensive or vasopressor medications before the onset of tension pneumothorax (or chronic use of antihypertensives); past history of hypertension, heart failure, or chronic pulmonary disease; and presence of a hemothorax or other pleural effusion, acute pulmonary disease (for example, pulmonary contusions), or pre-existing shock. Although we will also test whether differences exist in the reported risk of other clinical manifestations between patients of varying respiratory status, these analyses will be largely exploratory and more susceptible to type I error.

As the reported clinical manifestations of patients with a tension pneumothorax may be clustered within hospitals or treatment locations and are expected to occur relatively commonly, all dichotomous associations will be examined using generalized estimating equations with a log link, a binomial distributional family, and an independent within-group correlation structure [63]. These equations are a valid method for the analysis of common outcomes among correlated data, and can be used to adjust for the influence of data clustering when fitting multivariable binomial, logistic, and linear regression models [63]. Potential modifying or confounding variables included in the regression models will include those described above. If these models are unable to converge (as may occur when modeling the risk of an outcome), we will attempt to model the log of the odds using clustered logistic regression [63]. Finally, any potential differences in presenting systolic, diastolic, or mean arterial blood pressures (or differences in presenting systolic, diastolic, or mean arterial blood pressures from baseline) will be examined using generalized estimating equations with an identity link, a Gaussian distributional family, and a similar modeling strategy.

In order to determine the robustness of our findings, we will also conduct a series of sensitivity analyses, which will be reported alongside our principal results upon submission of the study findings for peer review. First, we will assess whether our observed associations are sensitive to incorporation bias or the classification of tension pneumothorax. After conducting analyses using the data extracted from all reported cases, this will be done by recalculating all outcomes using only those cases where two investigators independently agreed that the clinical condition of the study patient(s) aligned with the tension pneumothorax working definition described above [7,47]. Outcomes will also be recalculated using only those case reports and series where study authors gave absolute numbers rather than narrative or subjective descriptions when reporting the presence or absence of hypotension.

Second, as we anticipate that case reports and series may sometimes not report (or fail to clearly report) whether potentially important confounding/modifying or clinical manifestations data such as hypotension were specifically present or absent, we will also treat unclear/unreported data as missing (where they could not be clarified by writing study authors) and use imputation methods on these data. Multiple imputation is a method that may be used to perform a series of imputations for each missing observation by conducting random draws from the conditional distribution of the outcome variable given the values of the other variables [64]. After performing these imputations, regression analyses will then be conducted on each of the imputed data sets. The estimated associations and standard errors obtained from these analyses will then be combined to obtain point estimates and standard errors that account for the missing information. Simple imputation will then be used in order to provide an estimate of the extremes of influence of the missing values on the estimated outcomes between groups. This will be performed by first assigning all the missing values in the positive pressure ventilation group a value of ‘0’ and all the missing values for these variables in the breathing unassisted group a value of ‘1’. After recalculating the model with the inclusion of these imputed values, we will then reverse the assignment of the ‘0’ and ‘1’ values and then again recalculate the model in order to provide the opposite extreme estimate.

## Discussion

This study will provide the first systematic compilation of the world literature regarding the clinical manifestations of tension pneumothorax. As delayed or even missed diagnoses may result in poor outcomes and have been reported among patients lacking classically-described clinical manifestations of the disorder [7,18,19], an evidence-informed description of the clinical manifestations of tension pneumothorax may allow for creation of a list of evidence-based criteria for its diagnosis. Further, if our results support the pathophysiologic differences observed among animal studies of tension pneumothorax, it may also allow for the creation of separate definitions for the condition according to the presenting respiratory status of the patient. As delay in treatment of tension pneumothorax may adversely affect outcomes, and some clinicians may delay thoracic decompression among those suspected of having the condition as their hemodynamics are stable [65], this study could also potentially assist in identifying patients who may be appropriate candidates for treatment with needle or tube thoracostomy.

A specific objective of this study is to determine whether available literature supports a difference in reported presenting arterial blood pressures, time from suspected diagnosis to cardiac or respiratory arrest, and risk of hypotension and/or cardiac arrest among adults and children receiving positive pressure ventilation versus those that are breathing unassisted. We also aim to demonstrate our methods for the conduct of a narrative synthesis of systematic review results alongside a meta-analysis of observational studies and then a separate meta-analysis of case reports and series where appropriate. These include our proposed methods for the statistical combination of reported study results, including those that will be used to handle issues of confounding/modification and misclassified or unclear/unreported data among published case reports and series.

Although methods for the systematic review and meta-analysis of case reports and series have not yet been fully developed, these have been suggested [66,67], and are reportedly scheduled to be discussed for use in assessing the risk of rare adverse medication events at the next International Congress on Peer Review and Biomedical Publication [68]. However, in addition to their potential role in assessing the association between medications and rare adverse events [69,70], systematic reviews of case reports and series have also been utilized to evaluate the type, frequency of use, and effectiveness and safety of surgical interventions for conditions infrequently encountered in clinical practice [71,72]. As few other methods of study are sometimes feasible, these types of systematic reviews are also increasingly being used to evaluate the clinical manifestations or prognosis of rare, unusual, or difficult-to-study conditions [73-75]. In this study, using a rationale similar to that used by many of the above authors, we chose to include case reports and series as tension pneumothorax is a condition infrequently encountered in clinical practice and therefore difficult to study using an observational design.

However, while the Cochrane Collaboration and the Evidence-based Practice Center Program recognizes the usefulness of systematic reviews of case reports in select instances, the Cochrane Collaboration also expresses that several considerations must be made before their use in assessing adverse medication events [45,68]. Some of these considerations are also relevant to systematic reviews of case reports/series of clinical manifestations and therefore to this investigation, and will be addressed by our group using a number of complex evidence synthesis and analysis techniques. As no consensus definition for tension pneumothorax yet exists, it may be unclear as to the quality of the predictive value of the included case reports [45]. However, while it may be possible that we may somewhat over select for patients with less common etiologies of tension pneumothorax, we believe it would be less likely that their clinical manifestations would be substantially different than those with more common etiologies. Further, as tension pneumothorax is an uncommon yet catastrophic clinical diagnosis, its occurrence may be more likely to be reported regardless of cause, resulting in numerous case reports of tension pneumothorax among patients with more common etiologies (for example, central venous access punctures or lung disease).

As was also suggested by the Cochrane Collaboration, an adverse event (or in this case, clinical manifestation) is more plausible when a biological mechanism exists linking it to an intervention or exposure [45,68]. We believe our work to align with this consideration, as there now exists a considerable amount of preclinical data in support of a difference in pathophysiology between animals receiving positive pressure ventilation versus those who are breathing unassisted [7,17,22,23]. Moreover, although not outlined by the report from the Cochrane Collaboration, any association between the risk of certain clinical manifestations and the ventilatory status of the patient could be due to the presence of confounding factors. This outcome could also be influenced by the potential for clustering of clinical manifestations data in case series, a limitation that appears to have been relatively ignored by many previous meta-analyses of case reports and series data. In an attempt to address these issues, we outlined those factors that we felt *a priori* would be most likely to confound our relationships, and will attempt to adjust for the influence of these variables, and for any influence of data clustering, in our analyses.

## Conclusion

This systematic review will compile the world literature on tension pneumothorax and provide the first systematic description of its associated clinical manifestations to clinicians and other end users. As tension pneumothorax is frequently a difficult diagnosis that may be encountered in emergent situations [42], these data will be used to better inform health care providers on the clinical manifestations of the condition, and may contribute to an improved understanding of its appropriate definition, clinical diagnosis, and treatment. It will also demonstrate methods for the conduct of a narrative synthesis of systematic review results alongside a meta-analysis of observational studies and a meta-analysis of case reports and series. Results are expected to be publicly available in 2014.

## Abbreviations

ATLS: Advanced Trauma Life Support; FIO2: Fraction of inspired oxygen; ICU: Intensive care unit; MeSH: Medical Subject Heading; PaO2: Partial pressure of arterial oxygen; SpO2: Arterial oxygen saturation.

## Competing interests

The authors declare that they have no competing interests.

## Authors’ contributions

DR, SL-S, AK, JK, and HS conceived and designed the study. DR and PF designed the statistical analysis plan while DR, SL-S, and JK designed the search strategy, which was refined by HR. SL-S, PF, CB, HR, CB, ED, AK, JK, and HS provided input into the design of the study and draft of the protocol. DR wrote the first draft of the study protocol, which was critically revised by SS, PF, CB, HR, CB, ED, AK, JK, and HS. DR registered the protocol with the PROSPERO database. All authors read and approved the final protocol.

## Authors’ information

DR is a surgery and Clinician Investigator Program resident who is currently conducting a Doctor of Philosophy degree at the University of Calgary with a specialization in epidemiology. SL-S works as an Emergency Physician in the United Kingdom and has an interest in the pathophysiology and clinical manifestations of tension pneumothorax. PF is an academic biostatistician with an interest in complex data analysis while HR is an information scientist/medical librarian at the University of Calgary who specializes in systematic reviews. CB is a surgery resident who is currently conducting a Masters degree in medical education at the University of Calgary. CB, ED, AK and JK are academic trauma and/or general surgeons at the same institution who are actively engaged in research relating to elective or emergent surgical or critical care conditions. HS is an academic intensive care physician who has an interest in health services research related to emergent medical and surgical problems, especially those encountered in an intensive care unit setting.
